# Sustained ventricular arrhythmia and sinus node dysfunction revealing a cardiac amyloidosis: A case report

**DOI:** 10.1016/j.amsu.2022.104888

**Published:** 2022-11-12

**Authors:** Achraf Machraa, Walid Ben Brahim, Oussama Sidaty, Rokaya Fellat, Nadia Fellat

**Affiliations:** Dept. of Cardiology A – National Cardiovascular League, Ibn Sina University Hospital Center, Mohammed V University, Rabat, Morocco

**Keywords:** Cardiac amyloidosis, Infiltrative cardiomyopathy, Ventricular arrhythmia, Implanted cardioverter-defibrillator, Sinus node dysfunction

## Abstract

**Introduction and importance:**

Amyloidosis is an infiltrative multisystem disease due to extracellular deposition of fibrils in tissues and organs. Cardiac involvement can result in progressive heart failure, conduction abnormalities and arrhythmias and is associated with a poor prognosis. Atrial arrhythmias and non-sustained ventricular arrhythmias are the most common arrhythmias in cardiac amyloidosis. However, the association of sinus node dysfunction and sustained ventricular arrhythmia is quite exceptional.

**Case presentation:**

A 59-year-old male patient was admitted with a gradually worsening dyspnea with a lipothymic discomfort. Upon emergency department, an initial electrocardiogram revealed a severe bradycardia related to a sinus node dysfunction. A transthoracic echocardiography and cardiac magnetic resonance imaging showed features suspicious for cardiac amyloidosis. The diagnosis of AL cardiac amyloidosis with multiple myeloma was confirmed based on histological evidence. During hospitalization, the patient presented a sustained unstable ventricular tachycardia which has been converted by electrical cardioversion. He was treated with an implantable cardioverter-defibrillator (ICD) for secondary prevention with one episode of appropriate therapy. Unfortunately, the patient died few weeks later.

**Clinical discussion:**

The AL subtype of cardiac amyloidosis is associated with higher rates of arrhythmias, especially VT. The management of arrhythmias in cardiac amyloidosis is complex and remains challenging given the lack of evidence. ICD was not associated with longer survival; these findings underscore the importance of careful patient selection for ICD.

**Conclusion:**

As prognosis improves with the advances made in the medical treatment of cardiac amyloidosis, further studies are required to guide the management of all types of arrhythmias in cardiac amyloidosis.

## Learning points

• Ventricular arrhythmias and conduction system disease appear to be common in cardiac amyloidosis and carry prognostic implications
• ICD implantation for both primary and secondary prevention in cardiac amyloidosis has not been strongly supported by expert guidelines
• further studies are required to determine the optimal strategies to manage all types of arrhythmias since prognosis improves with the advances made in the medical treatment

## 1 Introduction

Cardiac amyloidosis is a progressive infiltrative cardiomyopathy that is caused by the deposition of misfolded fibrillar proteins in the extracellular space. Most of currently diagnosed cardiac amyloidosis due to monoclonal immunoglobulin light chain (AL) or transthyretin (ATTR) [1]. These depositions cause restrictive cardiomyopathy and lead to ventricular function deterioration.

Clinical manifestations of cardiac amyloidosis include diastolic heart failure secondary to restrictive cardiomyopathy, and a variety of arrhythmias.

Atrial arrhythmias and non-sustained ventricular arrhythmias are the most common arrhythmias in cardiac amyloidosis, while sustained ventricular arrhythmias are quite uncommon [2]. Here we report a case of AL cardiac amyloidosis in a patient with the association of sustained ventricular tachycardia and sinus node dysfunction.

This case report has been reported in line with the SCARE Criteria [3].

## 2 Case presentation

A 59-year-old man with a history of smoking (10 pack-years) presented to the emergency department for a progressive and disabling New York Heart Association (NYHA) III stage dyspnea with no associated chest pain. He did also report a lipothymic discomfort episode on minimal exertion. He was then admitted to the cardiac care unit for further investigation.

Physical examination revealed severe bradycardia (30 beats per minute), blood pressure of 120/90, there was a 2/6 systolic murmur in aortic area and no murmur detected in the carotid artery, vesicular murmur with basal crackles on pulmonary auscultation, his jugular venous pressure was elevated with a right hypochondrial pain.

**Fig. 1:**
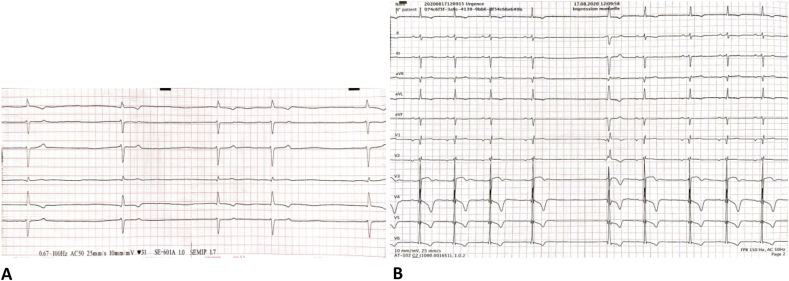
**A.** Limb leads: Admission ECG showing junctional bradycardia (30bpm) **B.** ECG showing sinus bradycardia, LAFB, PR = 200 ms, low voltage in the limb leads, sinus pause of 2s.

Electrocardiogram (ECG) revealed junctional bradycardia with 30bpm (Fig. 1A) that can be related to a sinus node dysfunction. Shortly after, another ECG showed a sinus bradycardia (45-50bpm), with a left axis due to left anterior fascicular block (LAFB), PR interval = 200 ms, low voltage QRS in the limb leads, T wave inversion in the lateral leads, and a sinus pause of 2s (Fig. 1B).

Laboratory tests revealed a slightly elevated potassium levels (5.7mmol/l), impaired renal function (eGFR = 18ml/mn/1,73m^2^) with increased levels of cardiac biomarkers [BNP = 3300pg/ml (reference value < 100pg/ml – Troponin: 143ng/l (reference value < 17ng/l)] which remained consistent in successive measurements.

**Fig. 2:**
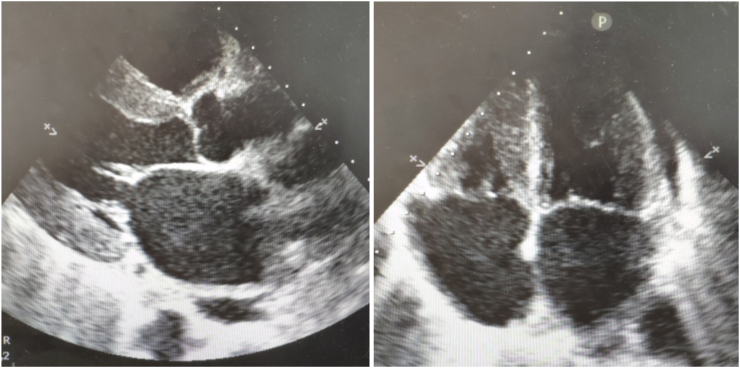
Echocardiography: Parasternal long axis view (A) Four chamber view (B) demonstrating increased thickness with a sparkling granular appearance of the myocardium, bi-atrial dilatation.

Echocardiography (Fig. 2) showed marked concentric left ventricular hypertrophy (Interventricular septum = 13mm – Posterior wall = 13mm) with a speckled appearance of the interventricular septum, bi-atrial dilatation, left ventricular ejection fraction of 65%, a small circumferential pericardial effusion. Doppler flow showed evidence of diastolic dysfunction. Coronary angiography was performed excluding the presence of coronary artery disease.

Taking the whole clinical picture into account, a suspicion of cardiac amyloidosis was made. Cardiac MRI was performed showing concentric hypertrophy predominant on the septal wall, the ejection fraction was 64%, there was subendocardial late gadolinium enhancement predominant on the septal and anterior wall compatible with cardiac amyloidosis.

**Fig. 3:**
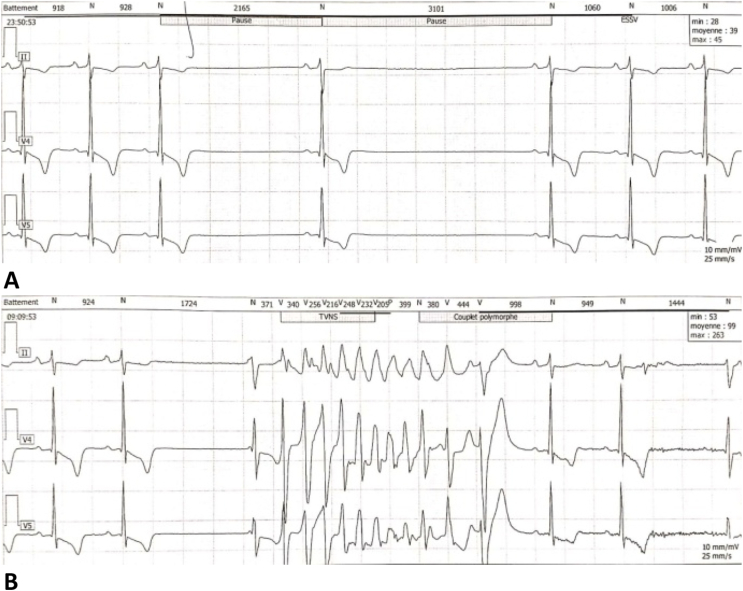
24 hours Holter ECG demonstrating the association of sinus node dysfunction (Panel A) with non-sustained VT (Panel B).

The holter ECG revealed the association of non-sustained ventricular tachycardia (VT) with a sinus node dysfunction (Fig. 3) requiring an implantable cardioverter-defibrillator (ICD).

**Fig. 4:**
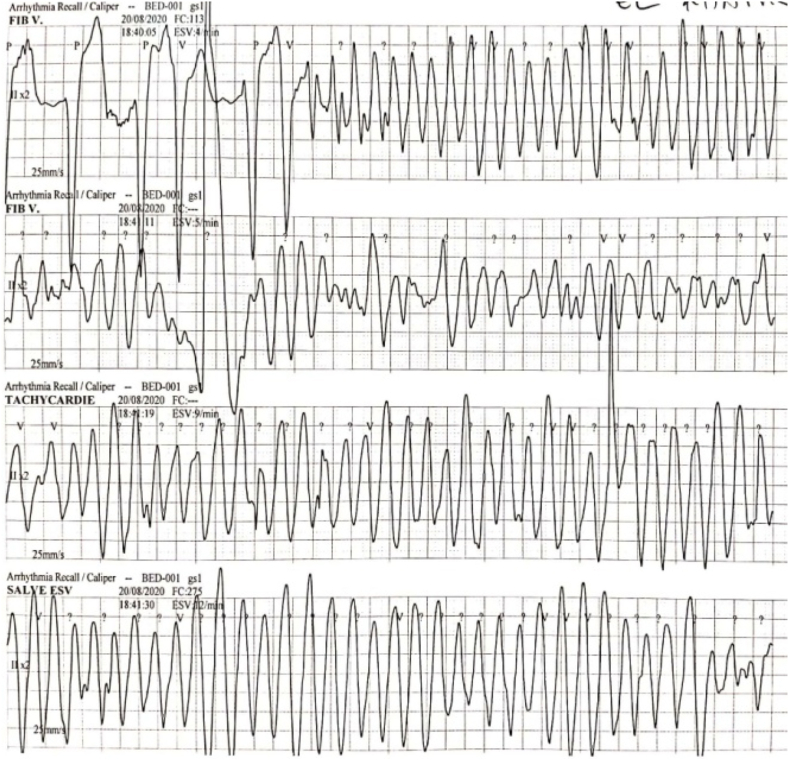
Monitoring trace showing the second episode of polymorphic VT after temporary pacing, this ECG also show the R on T phenomenon.

**Fig. 5:**
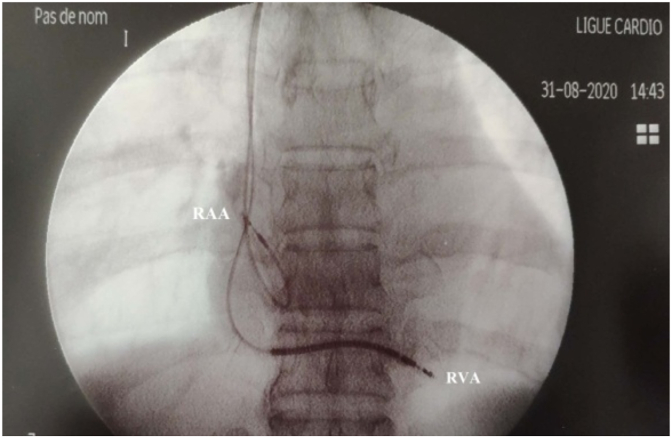
Fluoroscopic image in anterior-posterior view showing right placement of the right atrial and ventricular lead (RVA: Right ventricular apex/RAA: Right atrial appendage).

During hospitalization, the patient presented an episode of sustained polymorphic ventricular tachycardia with hemodynamic instability which was successfully terminated with 200J biphasic cardioversion. The ECG performed after tachycardia termination showed junctional bradycardia with polymorphic ventricular extrasystole, for which he was managed medically, and transvenous right ventricular apical temporary pacing was done. The next day, the patient suddenly developed a second episode of polymorphic VT (Fig. 4), it was managed with 200J biphasic cardioversion followed by intravenous amiodarone infusion. The decision to proceed with an ICD was made which was successfully done (Fig. 5). The initial outcome was favorable, the patient was asymptomatic.

Since cardiac amyloidosis was highly suspected. Minor salivary gland biopsy confirmed the presence of amyloid deposits, exhibiting green birefringence after staining with Congo red. A serum protein electrophoresis was then conducted to investigate a potential underlying gammapathy, a monoclonal protein was detected and confirmed with immunofixation. The monoclonal protein was defined as IgA-Lambda with a concentration of 731mg/l (Ratio Kappa/Lambda <0.10). After discussion with the hematology department, bone marrow biopsy was performed, which showed 67% involvement by abnormal appearing plasma cells.

**Fig. 6:**
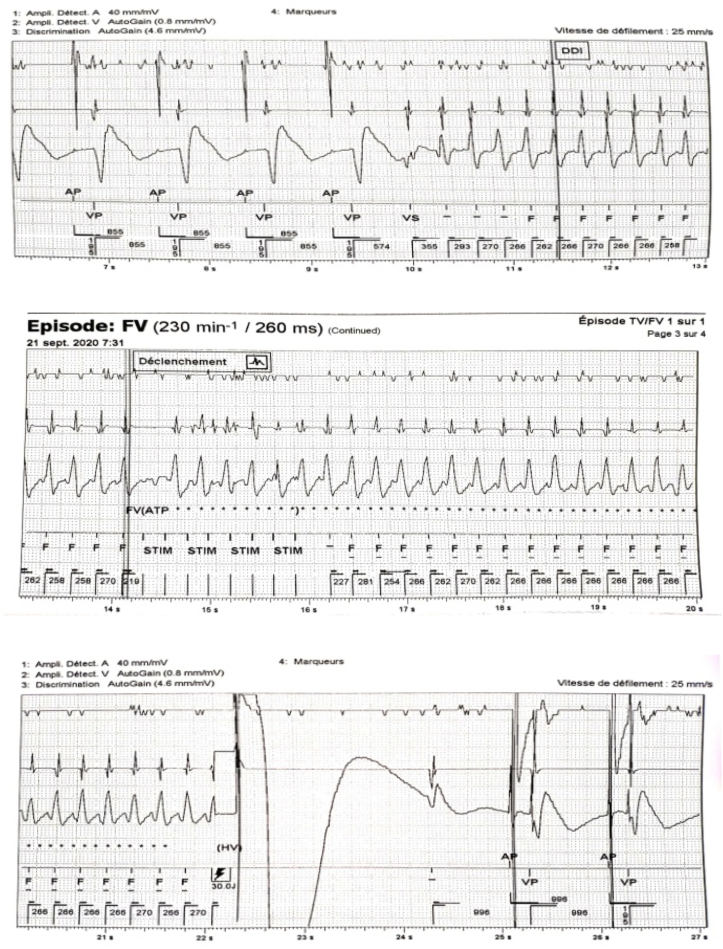
Intracavitary registration of a ventricular fibrillation (A) treated by the cardioverter-defibrillator with a shock (B). Subsequent restoration of electro-induced rhythm.

Based on the above findings, the diagnosis of cardiac amyloidosis secondary to multiple myeloma was made. Few days following placement, the ICD successfully intervened in one episode of ventricular fibrillation (Fig. 6).

Unfortunately, after a few weeks, the patient died before initiating systemic chemotherapy.

## 3 Discussion

Amyloidosis is an infiltrative multisystem disease due to extracellular deposition of fibrils in tissues and organs. Immunoglobulin light-chain (AL) amyloidosis - which is mainly related to monoclonal gammopathy of undetermined significance (MGUS) or myeloma - and transthyretin amyloidosis account for the vast majority of cardiac amyloidosis (CA) cases [1]. Cardiac involvement can result in progressive heart failure, conduction abnormalities and arrhythmias and is associated with a poor prognosis.

In cardiac amyloidosis, ventricular arrhythmias seem to be frequent and carry worse prognosis [4]. AL amyloid, with its more poor prognosis after the onset of heart failure symptoms, has a higher rate of ventricular arrhythmia compared with that of ATTR disease. The presence of non-sustained or sustained ventricular tachycardia on cardiac monitoring in patients with cardiac amyloidosis is associated with arrhythmic sudden cardiac death [5].

Available guidelines regarding ICD implantation in cardiac amyloidosis, either for primary or secondary prevention, are very limited for mainly two reasons [6]: 1) Electromechanical dissociation or agonal bradycardia from end-stage heart failure represent the most frequent documented mechanism of sudden death in CA [7,8]; 2) The worst outcome and low life expectancy in this population. As a result, there has been very little enthusiasm for the ICD in patients with CA. Based on such limited data, The current guidelines [9] consider that ICD implantation should be considered in secondary prevention, in patients with a life expectancy > 1year and good functional status, but predictors of survival in patients with CA and ICD are very poorly understood.

**Fig. 7:**
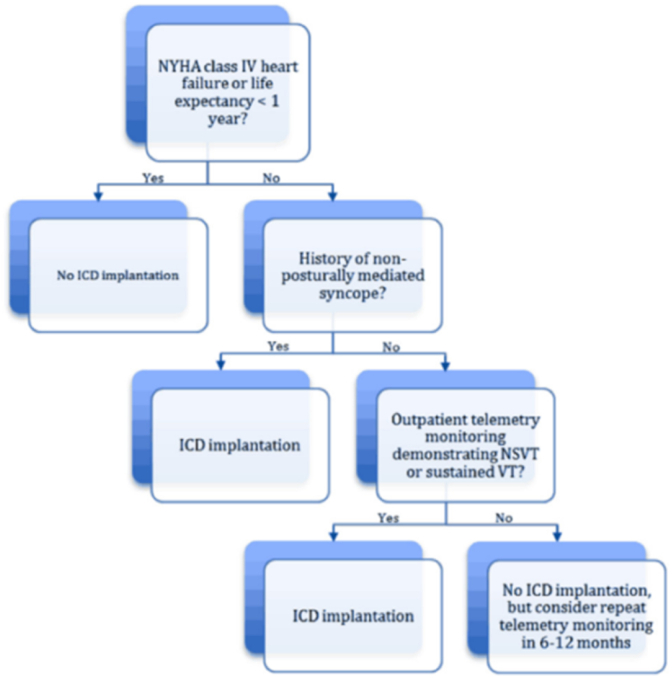
Stanford amyloid center's ICD implantation criteria in secondary prevention.

Available evidence however supports the use of Stanford Amyloid Center's ICD implantation criteria (Fig. 7) in secondary prevention [10].

Lina et al. [11] reported that there was no survival benefit in those with an ICD despite a high rate of appropriate ICD therapies. There are some reasons why an ICD may have limited efficacy for improving survival in CA, while ventricular arrhythmias are a common cause of sudden death in most cardiomyopathies, terminal bradycardia, or electromechanical dissociation is more frequent finding in CA [7].

In the present case, the device registered and effectively precluded an episode of ventricular fibrillation. Despite ICD implantation, mortality remains relatively high in patients with CA. These findings underscore the importance of careful patient selection for ICD, with close follow up after implantation.

Another point worth discussing is the management of conduction disease. The conduction system is affected in all forms of CA. Atrioventricular (AV) conduction delay or block appears to be more frequent than sinus node disease [12,13]. Conduction system abnormalities are a potential complication of amyloidosis due to amyloid deposits infiltration of the conduction system. Current guidelines recommend pacemaker implantation in patients with CA in the presence of advanced conduction disease.

## 4 Conclusion

Rhythm management in cardiac amyloidosis remains challenging given the lack of evidence. Sudden death and ventricular arrhythmias are frequent in cardiac amyloidosis, however, ICD implantation may not improve survival.

As prognosis improves in this population with the advances made in the medical treatment of cardiac amyloidosis, further studies are required to guide the management of all types of arrhythmias in cardiac amyloidosis.

## Ethical approval

Not applicable.

## Sources of funding

The authors declare that this work was not supported by any grants or funding support.

## Author contribution

Achraf Machraa was involved in the study concept, the collection of the data, drafting, literature review, and editing of the manuscript.

Walid Ben Brahim was responsible for literature review and revising the manuscript for important intellectuel content.

Oussama Sidaty was responsible for literature review and revising the manuscript for important intellectuel content.

Nadia Fellat was responsible of data validation and supervision.

Rokaya Fellat was responsible of data validation and supervision.

## Consent for publication

Written informed consent was obtained from the patient for publication of this case report and accompanying images. A copy of the written consent is available for review by the Editor-in-Chief of this journal on request.

## Registration of research studies

This is not and original research project involving human participants in an interventional or an observational study but a case report, this registration was not required.

## Guarantor

Achraf Machraa.

## Availability of data and materials

The data is available for sharing.

## Provenance and peer review

Not commissioned, externally peer-reviewed.

## Declaration of competing interests

All the authors declare that they have no competing interests.
